# The Importance and Control of Low-Grade Inflammation Due to Damage of Cellular Barrier Systems That May Lead to Systemic Inflammation

**DOI:** 10.3389/fneur.2019.00533

**Published:** 2019-05-29

**Authors:** Cecilia Rönnbäck, Elisabeth Hansson

**Affiliations:** ^1^Department of Ophthalmology, Rigshospitalet, Glostrup, Denmark; ^2^Faculty of Health and Medical Sciences, University of Copenhagen, Copenhagen, Denmark; ^3^Department of Clinical Neuroscience, Institute of Neuroscience and Physiology, The Sahlgrenska Academy, University of Gothenburg, Gothenburg, Sweden

**Keywords:** blood-brain barrier, blood-retinal barrier, blood-nerve barrier, blood-lymph barrier, blood-cerebrospinal fluid barrier, low-grade inflammation, systemic inflammation

## Abstract

Systemic low-grade inflammation can be initiated *in vivo* after traumatic injury or in chronic diseases such as neurodegenerative, metabolic, and autoimmune diseases. Inducers of inflammation trigger production of inflammatory mediators, which alter the functionality of tissues and organs and leads to harmful induction of different barrier systems in the body, where the blood-brain barrier, the blood-retinal barrier, blood-nerve barrier, blood-lymph barrier and the blood-cerebrospinal fluid barrier play major roles. The different barriers are unique but structured in a similar way. They are equipped with sophisticated junctional complexes where different connexins, protein subunits of gap junction channels and hemichannels, constitute important partners. The cells involved in the various barriers are coupled in networks, are excitable but do not express action potentials and may be targets for inflammation leading to changes in several biochemical cellular parameters. During any type of inflammation barrier break-down is observed where any form of injury can start with low-grade inflammation and may lead to systemic inflammation.

## Introduction

Neuronal signaling in the central nervous system (CNS) requires a balanced and well-controlled composition of the microenvironment around glial cells, neurons, and synapses. Loss of optimal function in the different barriers changes the vascular permeability, resulting in an increased transport of molecules and cells through the tight junctions into immune-privileged sites, resulting in destructive inflammation, as observed in many diseases. The body is composed of different barrier systems, including the blood-brain barrier (BBB), the blood-cerebrospinal fluid barrier (BCSFB), the blood-retinal barrier (BRB), the blood-nerve barrier (BNB), the endothelial-cell barrier (ECB), and the blood-lymph barrier (BLB). They are all responsible for preventing foreign matter penetration and protecting normal cell signaling.

The BBB controls the molecular mechanisms and the transport of molecules and cells between the blood and brain (1, 2). The BCSFB, a barrier including epithelial cells of the choroid plexus and the arachnoid epithelium as well as tanycytes, prevents fluctuations between the blood and the cerebrospinal fluid (CSF) (3). The BRB has well-developed tight junction complexes that maintain control of proteins and metabolites, which can enter the neural tissue (4). The BNB stabilizes the microenvironment around the endothelium of endoneural vessels, and layers traversing the perineurium regulate the exchange of materials via the blood-nerve exchange (5). The ECB is a barrier between the vascular lumen and the vascular wall that controls vascular tone and permeability and seems to be important in vascular inflammation (6). Finally, disturbances in the BLB result in infiltration of lymphoid tissue in different organs (7).

The present review highlights the different barrier systems and especially substances, molecules and cells that can affect and damage their protective mechanisms. Different types of cellular signaling can be disturbed, which is important in low-grade inflammation that can establish itself and lead to systemic inflammation. The brain and the nervous system play a central role because they can sense all changes that occur in different types of inflammatory processes.

## Low-Grade Inflammation Can Lead to Systemic Inflammation

Inflammation plays a major role in preserving physiological homeostasis of an organism and is initiated when pathogens, bacteria, viruses, etc. are presented. Inflammation is first initiated on a cellular level, low-grade inflammation, and can from there expand to inflammation involving different inflammatory cascades and organs causing systemic inflammation. Inflammation plays a central role in the body homeostasis and will, protect the body from illness and disease. On the other hand, prolonged or chronic inflammation releases proinflammatory cytokines and may expose the body to unfavorable conditions (8, 9).

The vertebrate inflammatory system is composed of the innate and adaptive inflammatory systems, and both are equally important. The innate immune system consists of macrophages, dendritic cells, mast cells, etc. Its main function is to detect bacteria, viruses, and foreign bodies in the organism as well as to activate the adaptive immune system and complement cascades. The adaptive immune system is highly specific and has developed throughout our lives. It consists of T- and B-lymphocytes, where pattern recognition receptors, including toll-like receptors, along with the induction of cytokines, play a major role in the activation of the adaptive immune system. Cytokines, consisting of small proteins, are produced by a variety of cells, including T- and B-lymphocytes. Cytokines can be proinflammatory or anti-inflammatory and may act as triggers for the release of other cytokines. They are important in chronic inflammation caused by oxidative stress (10).

In recent years, research has begun to focus on the roles of gap junction coupled cells which form networks in different organs in the body. Examples of cells coupled into networks include astrocytes, keratinocytes, chondrocytes, synovial fibroblasts, osteoblasts, connective tissue cells, cardiac and corneal fibroblasts, myofibroblasts, hepatocytes, and different types of glandular cells (11). They may be affected by different types of inflammatory stimuli, the cell signaling is changed through the connexin linked gap junctions (12), and the cellular networks become dysregulated (11). An underlying mechanism to an inflammatory response at the site of the damaged or affected nerve is the presence of a low-grade inflammation. Inflammatory substances such as histamine, bradykinin, 5-HT, glutamate, purines, tryptases, chymases, cytokines, growth factors, free radicals, nitric oxide (NO), etc are released from connective tissue cells such as macrophages and mast cells and are transported by the blood from the injured region and influence different barrier systems (13). Different inflammatory substances are also released from neurons located in the spinal cord and brain, which leads to an overactivation in the synaptic area. Resting microglia react and release cytokines. The astrocytes will then be reactive and can turn into dysfunctional astrocytes (1, 14). A low-grade inflammation can turn into a pathological state.

**Figure 1:**
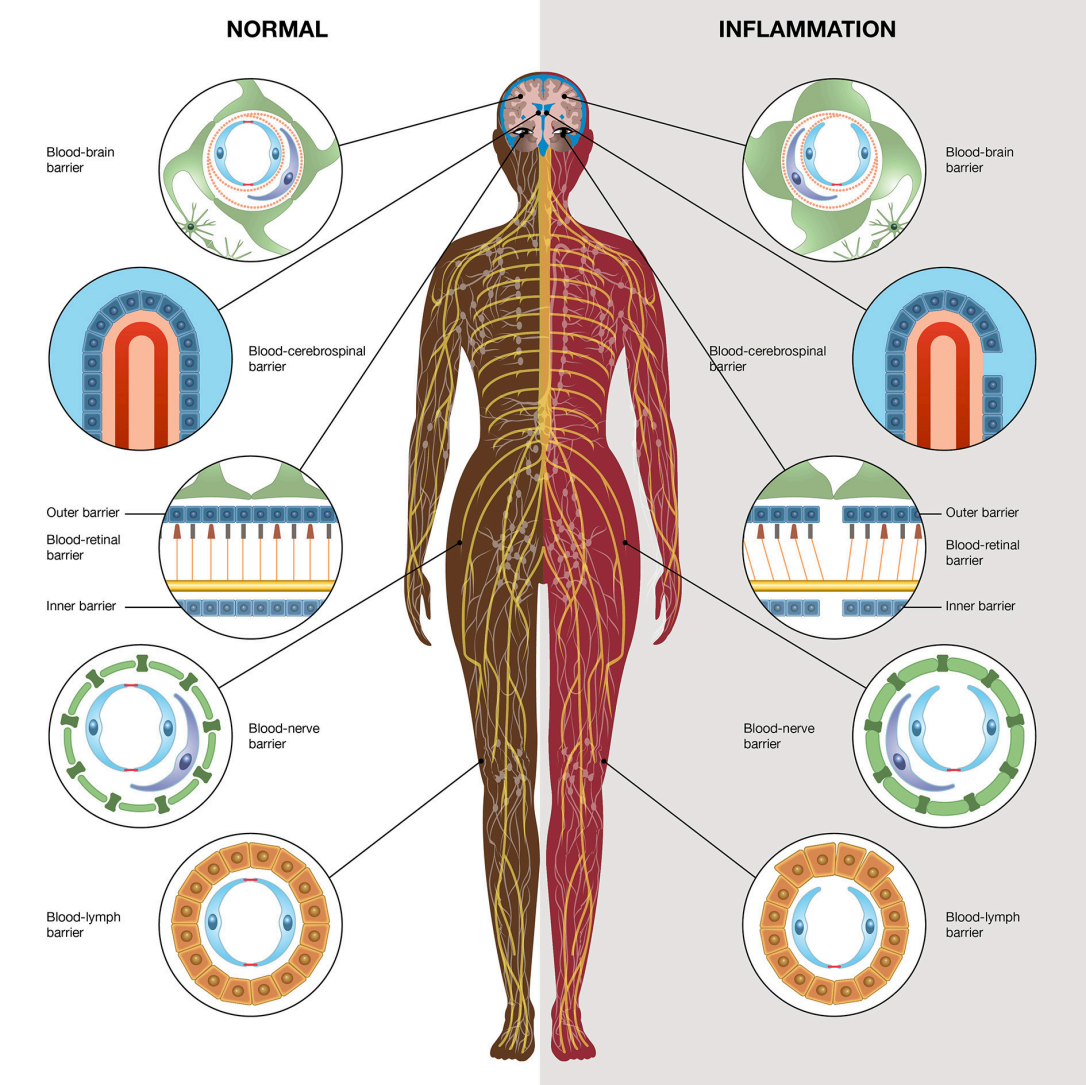
Schematic illustration high-lightening different barriers in the body; the blood-brain barrier, blood-retinal barrier, blood-nerve barrier, blood-lymph barrier, and blood-cerebrospinal fluid barrier. The left side demonstrates the normal physiological conditions and the right side demonstrates inflammatory conditions. The illustration is made by Pontus Andersson, ArtProduction, Gothenburg, Sweden.

Barriers at different sites in the body might be affected and can cause spread of inflammatory substances affecting other network-linked gap junction cells in other organs, which can give rise to systemic inflammation (Figure 1).

### Blood-Brain Barrier (BBB)

The BBB is located in the cerebral blood vessels. The blood vessels consist of endothelial cells and between them are tight junctions and adherens junctions, which limit the paracellular diffusion of solutes and ions between the blood and the brain (3, 15). Thus, transendothelial electrical resistance (TEER), which measures the resistance of tight junctions, is maintained by the components of the BBB. Tight junctions are composed of several proteins, among which the most prominent occludins and claudins are present (16). The vessels are surrounded by the basal lamina in which pericytes are found. The astrocytic perivascular endfeet rest against the basal lamina together with some microglia, and these cellular structures form the neurovascular unit (1). The BBB is controlled by intercellular signaling processes between different compartments that regulate the exchanges between the CNS and the blood. Small gaseous molecules, such as oxygen (O_2_), carbon dioxide (CO_2_) and nitric oxide (NO), diffuse through the membranes as well as small lipophilic agents. Transport systems on luminal and abluminal membranes regulate the traffic of small hydrophobic molecules. The combination of intra- and extracellular enzymes provides a metabolic barrier. The different BBB cell types and the molecular constituents have been thoroughly discussed recently (17). The brain is supplied with nutrients conveyed by endothelial transporters for glucose, amino acids, large neutral amino acids, and transporters for nucleosides, nucleobases, etc. (1). The astrocytic endfeet are enriched in water channel aquaporin 4 (AQP4) and in potassium channels as well as different cytoskeletal-associated proteins (16).

### Blood-Retinal Barrier (BRB)

Two BRBs exist: an inner and an outer BRB. The inner barrier controls fluid entry into the retina and is formed by retinal endothelial cells, and the outer barrier is formed by the retinal pigment epithelium, Bruch's membrane and the choriocapillaris (18). The endothelial cells of retinal vessels are equipped with tight junctions, adherens junctions, and gap junctions of retinal capillary endothelial cells. There are no fenestrations, and the TEER is similar to that in the BBB. The most prominent proteins in tight junction endothelial cells are occludins and claudins, but other proteins are present (18). The transport across endothelial cells in the inner barrier is regulated by membrane transporters and vesicular transport. At the abluminal side of the vascular retina, pericytes are present. They share the basement membrane together with the endothelial cells and contribute to the regulation of the inner barrier. The endfeet or processes from glial cells, astrocytes, Müller cells and microglia wrap around the vascular basement membrane of the retina, forming glial limitans (19).

The outer BRB is an intercellular junction complex created by tight junctions of retinal pigment epithelial cells that separates the neurosensory retina by the retinal pigment epithelium (RPE) from the blood supply of the choroidal circulation (20). Alterations and disturbance of the BRB are, among others, seen in diabetic retinopathy and age-related macular degeneration (AMD). Diabetic retinopathy is initiated by an alteration in the inner BRB (19, 21), and neovascular AMD is a result of an alteration in the outer BRB (22).

### Blood-Cerebrospinal Fluid Barrier (BCSFB)

The BCSFB encases the choroid plexus, the CSF-producing structure, and is localized within the brain ventricles. The barrier is formed by the epithelial cells of the choroid plexus as well as the arachnoid epithelium and tanycytes in the other parts that take care of the CSF (3). Between the endothelial cells from the capillaries and the epithelial cells, tight junctions and adherens junctions are the structural components that regulate of the influx and efflux of molecules across the BCSFB. It functions as an immunomodulatory gate.

### Blood-Nerve Barrier (BNB)

The endoneurial microenvironment supports the blood-nerve exchange composed of endoneurial vessels and the perineurium. Therefore, the endoneurial microenvironment is protected from substances that can be harmful to Schwann cells and axonal functions (5).

### Blood-Lymph Barrier (BLB)

Lymph-to-plasma exchange depends on the pore size of the capillary permeability and the resistance in the endothelial capillary wall (23).

### Low-Grade Inflammation Causes Barrier Damage

All barriers in the nervous system, as well as in the rest of the body, are important for maintaining physiological homeostasis. During any type of inflammatory process in the body, structural alterations occur, resulting in decreased TEER and increased permeability of the barriers (Figure 1; Table 1). Inflammatory mediators from different tissues and cells have direct access to the nervous system via the circulatory system via blood and lymph. Macrophages, microglia, astrocytes and the astrocytic endfeet are activated. Cells in the nervous system will thereby be negatively affected, allowing the production of cytokines such as tumor necrosis factor alpha (TNF-α), interleukin-1 beta (IL-1β), interleukin-6 (IL-6), reactive oxygen species (ROS), and NO, and the increased sensitivity for inflammatory receptors by upregulating the toll-like receptors (TLRs), which activate transcription factors such as nuclear factor-kappa B (NF-κB). Low-grade inflammation also leads to downregulation of the Na^+^/K^+^-ATPase pump and disturbed Ca^2+^ signaling through gap junction-coupled cells. The different barriers are dynamic structures where nutrients penetrate the barriers by passive diffusion or by transporters that supply the nervous system with necessary nutrients. Barrier disruption causes an increase in the migration of leukocytes through the barriers, and excessive ROS production occurs as well as the activation of matrix metalloproteases (MMPs) (1, 28).

**Table 1:** Characteristics for the blood-brain barrier, blood-retinal barrier, blood-nerve barrier, blood-lymph barrier and blood-cerebrospinal fluid barrier in physiological and inflammatory conditions.

**BLOOD-BRAIN BARRIER (BBB)**	
Cells and organelles:	Capillary endothelial cells, pericytes, perivascular endfeet of astrocytes, basal lamina, microglia, neuronal processes. Tight junctions between the endothelial cells (1, 2, 13).
Inflammatory changes:	Tight junction alterations, upregulation of GLUT1 transporter, increase of inflammatory mediators; histamine, bradykinin, 5-HT, glutamate, purines, cytokines, growth factors, complement-derived polypeptides, free radicals, NO, lipids etc., (1).
Barrier breakdown at pathological disorders:	Infectious or inflammatory processes; autoimmune diseases, MS, EAE, NMO; trauma; epilepsy; Parkinson's disease; neurodegenerative diseases, etc. see (1, 13).
**BLOOD-RETINAL BARRIER (BRB)**	
**Inner barrier**	
Cells and organelles:	Retina vessel endothelial cells, astrocyte endfeet, Müller glial cells, pericytes, smooth muscle cells. Tight and gap junctions between endothelial cells (14).
Inflammatory changes:	Tight junction alterations, transendothelial transport, changes of cells taking part in the barrier (14).
Barrier breakdown at pathological disorders:	Macular edema, hyperglycemia, diabetic retinopathy, oxidative stress (14).
**Outer barrier**	
Cells and organelles:	Retinal pigment epithelial cells. Tight, adherens and gap junctions between the epithelial cells, Bruch's membrane, choriocapillaris (14, 16).
Inflammatory changes:	Tight, adherens and gap junction alterations
Barrier breakdown at pathological disorders:	AMD (18).
**BLOOD-CEREBROSPINAL FLUID BARRIER (BCSFB)**	
Cells and organelles:	Cubodial choroid plexus epithelial cells. Tight, adherens and gap junctions between the epithelial cells (3).
Inflammatory changes:	Tight, adherens and gap junction alterations (3).
Barrier breakdown at pathological disorders:	Neurodegenerative diseases, cerebral amyloid angiopathy, ischemia, tumors, HIV (24, 25).
**BLOOD-NERVE BARRIER (BNB)**	
Cells and organelles:	Endothelial cells, endoneural space, schwann cells, fibroblasts collagen fibrils. Tight junctions between endothelial cells (5).
Inflammatory changes:	Tight junction alterations (5).
Barrier breakdown at pathological disorders:	Diabetic neuropathy, lead neuropathy (5)
**BLOOD-LYMPH BARRIER (BLB)**	
Cells and organelles:	Lymphatic endothelial cells, basal lamina. Tight junctions between the endothelial cells [(5, 26)].
Inflammatory changes:	Tight junction alterations (5).
Barrier breakdown at pathological disorders:	Inflammation, obesity, metabolic syndrome, inflammatory bowel disease (27).

*NO, nitric oxide; MS, multiple sclerosis; EAE, experimental autoimmune encephalomyelitis; NMO, neuromyelitis optica; AMD, age-related macular degeneration*.

The metabolic and energy state in the body is affected by dysregulation of glucose levels. A decrease in extracellular glucose leads to a reduced ability of ATP to properly maintain the activity of the Na^+^/K^+^-ATPase pump (29). Signaling systems between gap junction-coupled cells, such as astrocytes, are changed. This includes intercellular Ca^2+^ waves (30) and intercellular Na^+^ waves (31). If glucose uptake does not work properly, glucose degradation (glycolysis), an important function that supports signaling mechanisms in the brain, is reduced (32). This mechanism is important because lactate is formed from glycolysis and is utilized by neurons, playing an essential role in learning and learning-related long-term potentiation (LTP) (33). Glutamate induces glucose transporter (GLUT) activity and thereby uptake rates in astrocytes (34). For this astrocytic metabolism, which consumes ATP, Na^+^/K^+^-ATPase activity is increased. Increased lactate release as a result of increased glucose uptake and glucose flux through the glycolytic pathway might contribute to anti-inflammatory cellular actions by inhibiting glutamate release (35). Glucose transport in low-grade inflammation and in autoimmune diseases is changed. Organs other than the brain enhance glucose uptake, but brain cells react differently. Whether reduced glucose uptake is a result of reduced blood flow or insufficient glucose uptake due to subnormal glucose transport has been debated (36).

Low-grade inflammation can be initiated *in vivo* and can by damaged barriers and dysregulated gap junction coupled cellular networks spread by signals, today unknown, to other organs in the body. Signaling can spread or propagate from gap junction coupled cells in one organ to organs on either the contralateral or ipsilateral side (37). The complexity of actions evoked by endogenous or exogenous mediators has recently been discussed (38). During an inflammatory process a disruption of epithelial junctions can lead to increased transepithelial permeability by secreting pro-inflammatory cytokines or other inflammatory inducers that mediate epithelial barrier dysfunction (39). A consequence of inflammatory signaling is mucosal inflammation in the gut, airway epithelium, renal tubular epithelial cells, retinal epithelial cells, epithelium in the pancreas to mention some (39). Coexisting diseases, comorbidity, might be a consequence with barriers not working in a proper way (40). Metabolic disturbances can induce low-grade inflammation in all metabolically active organs such as the liver, adipose tissue and heart. Low-grade inflammation can occur in any organ which causes damage to various barriers. This in turn, can lead to comorbidity which can cause systemic inflammation.

### Network-Coupled Cells

Cells taking part in any barriers are connected to each other via gap junctions and thereby build networks (11, 41). Gap junctions are composed of members of the connexin family, which consists of proteins that play important functions in inflammatory diseases. Astrocytes, which are morphologically and functionally heterogeneous cells (42), play the greatest role in providing metabolic support for neurons and thereby represent a rate-limiting step in the complex homeostatic activity in brain health and disease. The intersignaling of small molecules through the supporting gap junction-coupled networks is accompanied by energy challenge and high energy consumption (29). The Na^+^/K^+^-ATPase pump detects the energy products in the glycolytic cycle where ATP plays a prominent role (29, 43, 44).

The connexin-based gap junction channels and the connexin-based hemichannels transmit electrical signals through their cellular networks to chemical synapses, which are regulated by neurotransmitters and second messenger pathways (45). These stimuli evoke intracellular increases in Ca^2+^ in single cells and passed to adjacent cells through connexin-based gap junction channels that can propagate intercellular Ca^2+^ signaling over long distances (46–48). Stimuli that evoke intracellular increases in Ca^2+^ concentration can also pass to adjacent cells through the connexin-based hemichannels via extracellular Ca^2+^ signaling (49). Intercellular and extracellular Ca^2+^ signaling result in the release of gliotransmitters such as glutamate, ATP and neuropeptides (50).

Upon inflammatory processes, astrocytes become reactive (51) and are affected by an overactivation of Ca^2+^ signaling that induces the release of ATP to the extracellular medium through connexin-based hemichannels (52). The released ATP activates the purinergic receptors in astrocytes, which further increases intracellular Ca^2+^ release. Therefore, downregulation of Na^+^ transporters and disruption of the cytoskeleton occur. Furthermore, proinflammatory cytokine release, neuronal excitability, and glutamate release all increase. Astrocyte uptake of excessive extracellular glutamate occurs through glutamate transporters, and ionotropic and metabotropic glutamate receptors are activated (14, 51, 53).

The plasma membrane can be discussed as a barrier because it is a dynamic entity that separates the extracellular and intracellular environments and is also a structure composed of proteins and lipids, which both control and are controlled by biological processes.

The actin cytoskeleton and microtubules are intimately associated with the plasma membrane (54). Inflammatory stimuli can trigger plasma membrane transduction and protein recruitment, which may result in alterations to the cytoskeletal structure. Actin is the most abundant cytoplasmic protein and regulates cellular and immune functions through a complex cytoskeleton-dependent process (55). Actin appears in two forms, globular actin (G-actin) and filamentous actin (F-actin), and the transition between these two forms is a dynamic process driven by polymerization and depolymerization (56). The cytoskeleton controls the plasma membrane microdomains and the endoplasmic reticulum complex. The adaptor protein ankyrin B is associated with Na^+^/K^+^-ATPase and with endoplasmic reticulum proteins such as the inositol 1,4,5-trisphosphate receptor (IP_3_). The main cytoplasmic matrix of proteins, spectrin and actin, are attached to ankyrin B. An intact cytoskeleton is required for the propagation of astrocytic Ca^2+^ waves (49), and cytoskeletal disruption abolishes Ca^2+^ oscillations by changing the balance between the Ca^2+^-regulating processes (57). Actin filaments are normally organized in stress fibers, but after exposure to inflammatory stimuli, the actin filaments can be disorganized and appear as ring structures (51, 53). During destruction and tissue remodeling caused by inflammation, the actin cytoskeleton plays an important role. The F-actin filaments may be responsible for the loss of junctions in inflamed cells due to the effects of microbial toxins and inflammatory mediators (39). Involvement of the cytoskeleton in controlling the plasma membrane microdomains and the endoplasmic reticulum complex seems to be important.

### Signaling That Affects Cellular Barriers

Searching for molecular mechanisms to explain how inflammatory processes develop in different cells, tissues and organs and how they can be spread through different systems may be one important task for the treatment of low-grade inflammation that can lead to prolonged pain. The extracellular matrix is an important part of all biological barriers, and it modulates the interchange through these barriers. It controls the migration of leukocytes from the blood, a process that is altered in inflamed tissues in which different extracellular matrix components are involved that influence the inflammatory response. The development of nanotechnology and the application of nanoparticles have led to new research aimed to solve the question of inflammatory processes (58). Endothelial cells with microvascular compartments are sensitive to signals generated in the blood and control the substances or particles that will pass different barriers.

Mast cells and glial cells show endogenous homeostatic mechanisms that can be upregulated in response to ongoing inflammation (59). Mast cells originate from the bone marrow and circulate in the blood as precursor cells. They complete their differentiation in tissues they are meant to stay in, on the abluminal side of blood vessels. Thereby, they can communicate with cells, the extracellular matrix, and blood vessels and probably have a large influence on different barrier systems. Mast cells can migrate from the blood to the brain through the barriers both during normal and pathological situations, thereby disrupting tight junction proteins (60). Additionally, a mast cell-glia communication exists (61). Mast cells can be the first responders to injury/inflammation because they are effector cells of the innate immune system. These cells produce a multitude of substances, including biogenic amines, cytokines, enzymes, lipid metabolites, ATP, neuropeptides, growth factors, NO, and heparin (59). The tryptase and chymase enzymes are the major proteins stored and can be released upon inflammatory signals, which can promote matrix destruction and tissue remodeling (13). The tryptase receptor proteinase-activated receptor 2 (PAR2), which is present on most supporting cells, including glial cells, is activated upon inflammation (59, 62). Nerve growth factor (NGF) is rapidly released during inflammation and activates its nociceptors tropomyosin-related kinase A (TrkA) and p75 neurotropin receptor present on most supporting cells, including glial cells. The cells respond in a paracrine/autocrine fashion to NGF and thereby promote the recruitment of other cells. The contribution of activated mast cells is complex because immediate activation can limit cellular disturbances and thereby brain damage as they produce cytotoxic molecules as well as growth and repair factors. An ongoing activation of mast cells can lead to more destructive changes.

## Conclusion

Functional barriers such as the BBB, BRB, BCSFB, BNB, and BLB enable physiological homeostasis within tissues and organs. The barriers can resist acute inflammation, while prolonged inflammation and oxidative stress may lead to systemic inflammation affecting the nervous system and retina, cartilage, blood vessels, and nerve endings. It is of great importance to understand the different barriers, their functionality and how they act. Although there are still many unsolved questions, understanding why some people are more affected by diseases than others are remains of critical importance.

## Author Contributions

All authors listed have made a substantial, direct and intellectual contribution to the work, and approved it for publication.

### Conflict of Interest Statement

The authors declare that the research was conducted in the absence of any commercial or financial relationships that could be construed as a potential conflict of interest.
